# Preparation of Linalool/Polycaprolactone Coaxial Electrospinning Film and Application in Preserving Salmon Slices

**DOI:** 10.3389/fmicb.2022.860123

**Published:** 2022-04-15

**Authors:** Tingting Li, Xinghui Zhang, Jialin Mei, Fangchao Cui, Dangfeng Wang, Jianrong Li

**Affiliations:** ^1^College of Life Science, Dalian Minzu University, Dalian, China; ^2^College of Food Science and Technology, Bohai University, Jinzhou, China; ^3^School of Food Science and Technology, Jiangnan University, Wuxi, China

**Keywords:** coaxial electrospinning, linalool, salmon, polycaprolactone, preservation

## Abstract

A linalool/polycaprolactone (LL/PCL) antibacterial film was prepared by using a coaxial electrospinning process, and its physical and chemical properties were characterized. The antibacterial film was formed into an active antibacterial gasket, and its effect on salmon preservation was analyzed. The results show that the LL/PCL nanofiber membrane had a well-developed microstructure, and the fiber surface was smooth and uniform. The diameter of the fibers in the PCL membrane without the core material (linalool) was 113.92 ± 23.74 nm. In contrast, the diameter of the coaxial nanofiber membrane with linalool increased, and the diameter of the LL/PCL membranes with 20% and 40% linalool was 220.62 ± 44.01 and 232.22 ± 56.27 nm, respectively. The hydrophobicity and water vapor permeability were enhanced, whereas the tensile strength and elongation at break decreased slightly, while the thermal stability did not differ significantly with the incorporation of linalool. Analysis of the sustained release of linalool showed that the LL/PCL coaxial fiber membranes could release linalool into the reaction system for a long time. The LL/PCL nanofiber film was used to create an antibacterial active gasket for salmon preservation experiments. Sensory evaluation and analyses of the total bacterial count, total volatile basic nitrogen (TVB-N), thiobarbituric acid reactive substances (TBARS), pH, texture (hardness, elasticity, chewiness, and viscoelasticity), water distribution change, and aroma using an electronic nose were used to determine the quality of salmon. It was found that food-grade tinfoil and the PCL gasket had no significant effect on the freshness of salmon, while the active antibacterial gasket samples containing linalool could decrease the rate of decay salmon and effectively prolong the shelf-life of salmon by releasing linalool.

## Highlights

- Linalool (LL) loaded polycaprolactone mats were obtained by coaxial electrospinning.
- LL-loaded fibers applied in active antibacterial gasket of salmon provided fresh keeping activity.
- LL can be released into the reaction system for a long time from coaxial fiber membranes.

## Introduction

Food packaging, one of the key components of food systems, serves the purpose of isolating food from external factors and ensuring food quality and safety. Social demand and industrial production trends have highlighted safety and environmental protection in the development of food packaging, as well as green, convenient, and intelligent production (Realini and Marcos, 2014; Han et al., 2018). Active food packaging is an innovative means of maintaining or extending the shelf-life of food by adding active ingredients while ensuring food quality, safety, and integrity (Biji et al., 2015; Vilela et al., 2018). By using active packaging instead of directly adding active substances, such as antibacterial agents and antioxidants to food, the amount of such substances can be greatly reduced. In addition, direct addition of active substances to food may lead to interactions between the active substances and food ingredients, or the efficacy of the active substances may be greatly reduced or inhibited due to subsequent food processing (Yildirim et al., 2018).

In recent years, the application of electrospinning technology in the preparation of active food packaging materials has become a research hotspot. The principle is that the charged jet forms a stable Taylor cone under the action of an electrostatic field force; the cone is transported to the receiving device, and finally forms micro/nanofibers (Bhardwaj and Kundu, 2010). Electrospinning technology has some outstanding advantages, such as low cost, high fiber yield, and large specific surface area, making it suitable for manufacturing different types of materials (Hadad and Goli, 2018; Kopp et al., 2020). Coaxial electrospinning technology is an improvement of the original spinning process, where a special nozzle structure is used to prepare the core-shell structure of the fiber; thus, unstable small molecular functional compounds can also be wrapped in the shell polymer, under the protection of electrospinning, greatly improving the functionality of fiber materials. Thus, electrospinning technology can be applied in a number of fields. Ding et al. (2019) prepared coaxial polylactic acid–propyl gallate electrospun fibers and studied their effect on chilled salmon filets. It was found that the electrospun fiber had a small diameter, smooth surface, and no obvious defects. The thermal stability and tensile strength of the fibers made them suitable for cold storage. During storage, the rate of deterioration of salmon muscle tissue was significantly reduced, and the electrospun fibers had a significant inhibitory effect on bacterial spoilage. Notably, the degradability of food packaging has always been a concern. Polycaprolactone (PCL) has been applied in the field of electrospinning because of its nontoxicity, harmlessness, good film-forming properties, hydrophobicity, biocompatibility, and biodegradability (Woodruff and Hutmacher, 2010; Dodero et al., 2020; Yang et al., 2020).

Plant essential oils are active components that can be extracted from plants. The oil type influences the mechanism of action of essential oils. Essential oils act on bacteria by degradation of the cell wall (Gill and Holley, 2006), destruction of the cell membrane structure (Ultee et al., 2002) and membrane proteins (Lambert et al., 2001), inhibition of quorum sensing, influencing the proton dynamic potential energy (Ultee and Smid, 2001), and inhibiting intracellular ATP synthesis (Burt, 2004). Linalool (LL) is one of the main components of certain aromatic plant essential oils such as lavender essential oil and camphor essential oil (Aprotosoaie et al., 2014). Linalool is often added to food as an aromatic and flavoring agent. In addition to its simple flavoring function, LL also has antibacterial (Hussain et al., 2008), anti-inflammatory, antitumor (Cheng et al., 2017), anti-hypertensive (Camargo et al., 2018), and preventive effects against neurodegenerative diseases (Alzheimer’s disease, etc.; Cardona, 2016; Guaqueta-Sabogal et al., 2016).

Salmon, which is delicious, rich in unsaturated fatty acids, and has a high protein content, low cholesterol, low calorie content, and other advantages, is recognized by consumers in various countries (Joaquín et al., 2018). However, due to its high protein and fat content, the quality of salmon will inevitably decline during storage and transportation. Therefore, new preservation methods are urgently needed to ensure food safety during storage and transportation.

In this study, LL/PCL nanofiber films are prepared by coaxial electrospinning with linalool as an antibacterial agent and PCL as a substrate and are applied to the preservation of salmon filets. The physical and chemical properties of the nanofiber membrane are characterized by a number of techniques, including field-emission scanning electron microscopy (SEM), Fourier transform infrared spectroscopy (FT-IR), tensile strength testing (TS), water vapor transmission rate (WVP) analysis, differential scanning calorimetry (DSC), and thermogravimetric analysis (TG). The hydrophobicity of the membrane is verified by measuring the water contact angle, and the kinetics of linalool release from the film are also tested. Studies on the effects of linalool on maintaining the freshness on salmon are lacking; therefore, the present research is of innovative significance for broadening the application scope of linalool.

## Materials and Methods

### Materials

LL (>98%) and PCL (*M_W_* = 80,000) were purchased from Shanghai Yuanye Biotechnology Co., Ltd. Salmon was purchased from an aquatic market in Dalian, the origin of which is the Faroe Islands. After being slaughtered, the salmon were frozen in liquid nitrogen and transported to Dalian by air transportation. *N,N*-dimethylformamide (DMF), dichloromethane (DCM), and other reagents were commercially available; PCA was purchased from Solarbio, and all solutions were prepared with deionized water.

### Preparation of Spinning Solution

PCL was dissolved in a mixture of 30% DMF and 70% DCM to form a PCL system with a mass fraction of 18%. The mixture was stirred for 3 h at room temperature until the PCL was completely dissolved and was used as a wall material. Linalool was dissolved in anhydrous ethanol and sonicated for 3–5 min; the concentrations of linalool were 0%, 20%, and 40%.

### Electrospinning Process

LL/PCL nanofiber membranes were prepared by electrospinning different concentrations of the core material (LL) with the wall materials. In the absence of the core material (LL concentration = 0), the process parameters were as follows: the positive voltage was 13 kV, the negative voltage was −1.6 kV, and the flow rate of the spinning solution was 0.30 mm/min. Coaxial LL/PCL nanofiber membranes were prepared with core material concentrations of 20% and 40%, where the process parameters were as follows: the positive voltage was 22 kV, the negative voltage was −1.7 kV, and the spinning fluid flow rates for the shell and core layers were 0.25 and 0.05 mm/min, respectively. The receiving distance was 18.0 cm, and the spinning temperature was 25 ± 1°C. The relative humidity was 50%. After the films were removed, they were dried at room temperature for 12 h and sealed.

### Characterization of Electrospun Fibers

#### Scanning Electron Microscopy

The fiber membrane was sprayed with gold, and the micromorphology of the fiber membrane was observed using SEM. One hundred nanofibers were randomly selected from the SEM images using Image J image analysis software to measure the diameter, and the average diameter and standard deviation were calculated.

#### Infrared Spectroscopy

The fiber membrane was cut up, mixed with potassium bromide (1:50), ground and pressed, and the infrared spectrum of the fiber membrane was determined by scanning in the wavelength range of 4,000–400 cm^−1^.

#### Differential Scanning Calorimetry

The fiber membrane samples (5.00 mg) were weighed accurately with an analytical balance, and the thermal stability of the membrane was measured under nitrogen environment using a differential scanning calorimeter. The scanning range was 20–250°C, the heating rate was 10°C/min, and the temperature was maintained constant for a time of 1 min.

#### Thermogravimetric Analysis

The fiber membrane samples (3.00 mg) were accurately weighed with an analytical balance. Thermogravimetric analysis was performed in the temperature range of 50°C–600°C, with a temperature gradient of 10°C/min, under nitrogen environment.

#### Surface Hydrophobicity

The surface hydrophobicity of the nanofiber membrane was characterized by static water contact angle measurements. The membrane was cut into a 20 mm × 40 mm spline and pasted on flat glass, and 3 μl of distilled water was dropped on the film at nine different positions. The static contact angle was measured using the tangent method.

#### Water Vapor Permeability

Anhydrous CaCl_2_ (3.000 g) was added to a weighing bottle and dried to constant weight by heating for 24 h in a 105°C oven (weighing error is within 0.002 g). The weighing bottle was sealed with a spinning membrane, and the initial weight was recorded. A beaker with saturated NaCl solution was placed at the bottom of the dryer to provide a constant relative humidity. After 48 h at room temperature, the weight was measured every 12 h. The water vapor transmission rate was calculated using the following formula:


(1) $$WVT=\frac{\Delta m}{A\times t}$$

where WVT is the water vapor transmission rate [g/(m^2^·d)], Δ*m* is the mass increment of the system (g), *A* is the effective area of the membrane (m^2^), and *t* is the determination time (d).

#### Mechanical Behavior

A micrometer was used to measure the thickness of the film prepared by 2 h spinning by randomly measuring five different points. The average value is reported as the thickness of the film. The sample was cut into a 7 cm × 3 cm rectangle using scissors, the edges of the sample were smoothed to remove notches, the two ends of the sample were fixed on the probe of the texture analyzer, the initial spacing of the sample was set to 10 cm, and the tensile speed of the texture analyzer was set to 10 mm/min. The average value of triplicate measurements (for three parallel samples of each type) is reported. The tensile strength (*Ts*) and elongation at break (*E_b_*) were calculated using the following formula:


(2) $$Ts=\frac{F}{bd}$$

where *F* is the maximum force (N) in the stretching process, *b* is the width of the sample (m), and *d* is the sample thickness (m).


(3) $$E_{b}=\frac{100\left(L_{b}-L_{0}\right)}{L_{0}}\times 100\%$$

where *E_b_* is the elongation at break, *L_0_* is the initial length of the sample (cm), and *L_b_* is the fracture length of the sample (cm).

### Slow Release Kinetics Test

Based on the slight solubility of linalool in water, an appropriate amount of linalool was dissolved in PBS buffer (pH 7.2–7.4), and full wavelength scanning was performed using a UV spectrophotometer. The maximum absorption appeared at 274 nm. Linalool (50 μl) was dissolved in 10 ml PBS buffer to form a 5-μl/ml linalool solution, which was then serially diluted to 2.5, 1.25, 0.625, 0.3125, and 0.1563 μl. The absorbance was measured at 274 nm using a UV spectrophotometer, and the standard curve of linalool dissolved in PBS buffer was plotted. Referring to the method of Guo et al. (2011), 20% and 40% linalool/polycaprolactone fiber membranes prepared with a spinning time of 1 h were placed in a conical flask filled with 50 ml PBS buffer, and the conical flask was placed on a shaking table at 37°C and agitated at 160 rpm for 3 days. The amount of linalool released from the fiber membrane was determined, and 3 ml samples were taken at different time points; the same amount of PBS buffer was added to the original release system to keep the concentrations unchanged.

### Evaluation of Salmon Freshness

A whole salmon was cut into 200 ± 5 g pieces after removing the head, skin, and bone. The samples were disinfected with alcohol and then irradiated with an ultraviolet lamp for 20 min. Antibacterial gaskets were made with different amounts of the LL/PCL coaxial electrospun fibers. The surface microorganisms were killed by irradiation with an ultraviolet lamp for 2 h. The sliced salmon filets were placed on a gasket and transferred into a sterile cooking bag. After sealing, the filets were stored in a refrigerator at 4°C for 20 days. Samples were taken every 4 days for the preservation test. A food tin foil gasket was used as a negative control, and a PCL fiber gasket was used as a positive control.

#### Colony Count

According to the method of Hu et al. (2017), the bacterial count was determined in triplicate (using three parallel samples) for each sample.

#### pH

For pH analysis, 5.0 g of sample was weighed, 45 ml of distilled water was added and homogenized, and the mixture was allowed to stand for 30 min. The pH of the supernatant was determined in triplicate (with three parallel tubes) for each sample.

#### Total Volatile Basic Nitrogen

The FOSS Kjeldahl nitrogen analyzer was used to determine the total volatile basic nitrogen (TVB-N) value in triplicate (three parallel samples) for each 5.0 g sample.

#### Thiobarbituric Acid Reactive Substances

For TBARS determination, the method described by Barbosa-Pereira et al. (2013) was applied with slight modifications. The samples (10.0 g) were added to 50 ml of 5% trichloroacetic acid, homogenized, and filtered. The primary filtrate was discarded, and 5 ml of supernatant was taken and reacted with 5 ml of 0.02/L thiobarbituric acid (TBA) solution in a water-bath at 80°C for 40 min. After color development, the sample was cooled and the absorbance at 532 nm was measured. The TBARS value is expressed as the mass fraction of malondialdehyde (MDA).

#### Texture Profiling Analysis

According to the method of Einen and Thomassen (1998), the fish was cut into small pieces (1.5 cm × 1.5 cm × 1.5 cm), and the samples were analyzed twice by compression TPA. The pre-test rate was 35 mm/s, the test speed was 1 mm/s, the recovery time was 2 s, the compression degree was 50%, the residence time was 5 s, and the trigger force was 5 g. The probe type was p/50, and the measurement indexes included the hardness, elasticity, chewiness and adhesiveness.

#### Determination of Moisture Distribution

The fish was cut into thin strips (1.0 cm × 1.0 cm × 3.0 cm) and placed at the bottom of the coil tube of the nuclear magnet. The nozzle was wrapped with a preservative film and kept at room temperature for 20 min. After equilibrating the sample temperature with the ambient temperature, the sample was placed in the probe coil for determination.

#### Electronic Nose

The minced salmon sample (10 g) was placed in a 100-ml beaker, sealed with three layers of preservative film, and then allowed to stand at 4°C for 60 min. An electronic nose system was used to detect the headspace gas in the beaker. Detection conditions: the sensor cleaning time was 100 s, the determination time was 120 s, and the injection flow rate was 300 ml/min.

### Statistical Analysis

Statistical analysis was performed using SPSS 19.0, and Origin 8.5 (IBM software, Chicago, United States) was used to generate the required charts.

## Results and Discussion

### Microstructure Analysis

Many factors influence the microstructure of electrospun fibers, including the solution viscosity, spinning distance, positive and negative voltage, and flow rate. The results of this experiment are as follows: solutions with low viscosity generally led to an uneven fiber, which was characterized by a large number of bead structures. The viscosity of these solutions is not suitable because the resulting fibers will have a negative effect on the process performance of the system. When the spinning distance was too large or too small, it was difficult to form a stable Taylor cone, and a well-structured fiber membrane was not obtained; when the spinning voltage was too large, branches were formed, and small droplets precipitated due to the insufficient electric field force. At very high flow rate, it was difficult to completely volatilize the solvent, whereas when the flow rate was too low, a continuous fiber was not obtained.

**Figure 1:**
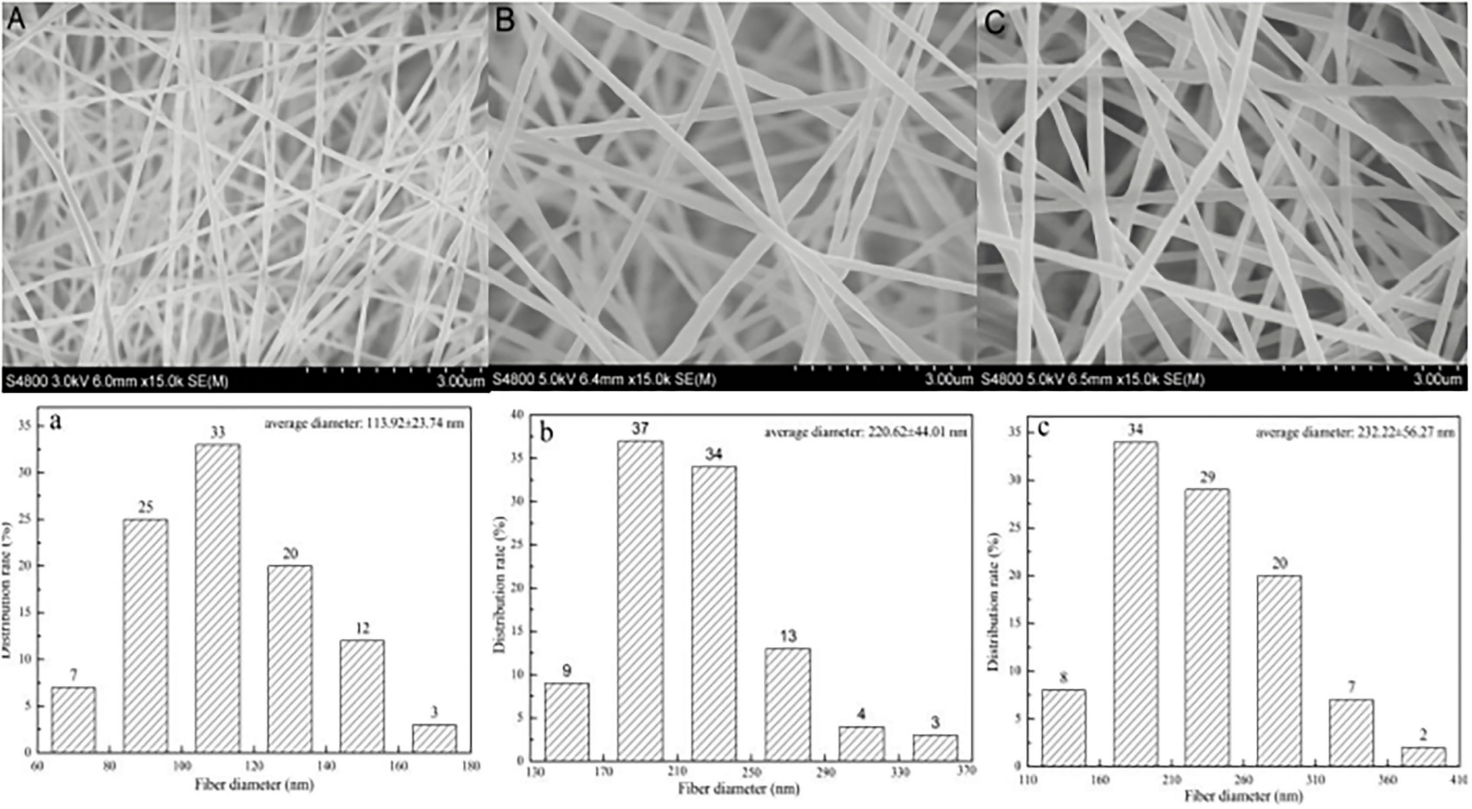
SEM images of **(A)** PCL fibrous membrane, **(B)** 20% LL/PCL fibrous membrane, **(C)** 40% LL/PCL fibrous membrane; and fiber diameter distribution of **(a)** PCL fibrous membrane, **(b)** 20% LL/PCL fibrous membrane, **(c)** 40% LL/PCL fibrous membrane.

Figure 1 shows the SEM image and diameter distribution of the linalool/polycaprolactone fiber membrane. The figure demonstrates that PCL has excellent spinning performance. As shown in Figure 1A, the uniaxial electrospun PCL nanofiber had a dense multilayer network structure, the fiber surface was smooth with no beaded structures, and the fiber diameter was evenly distributed between and 60–180 nm, with an average diameter of 113.92 ± 23.74 nm. The coaxial LL/PCL fibers are similar to uniaxial fibers, as shown in Figures 1B,C, but the fiber diameter increased significantly with addition of the active agent (LL), where the average diameter of the 20% LL/PCL fiber membrane was 220.62 ± 44.01 nm. The diameter of the membrane fiber changed from 44.01 to 232.22 ± 56.27 nm with 40% LL concentration, which indicates that the agent was well embedded in PCL. This has a certain protective effect on linalool, the addition of which increases the diameter of the polymer spinning fiber.

### Infrared Spectral Analysis

**Figure 2:**
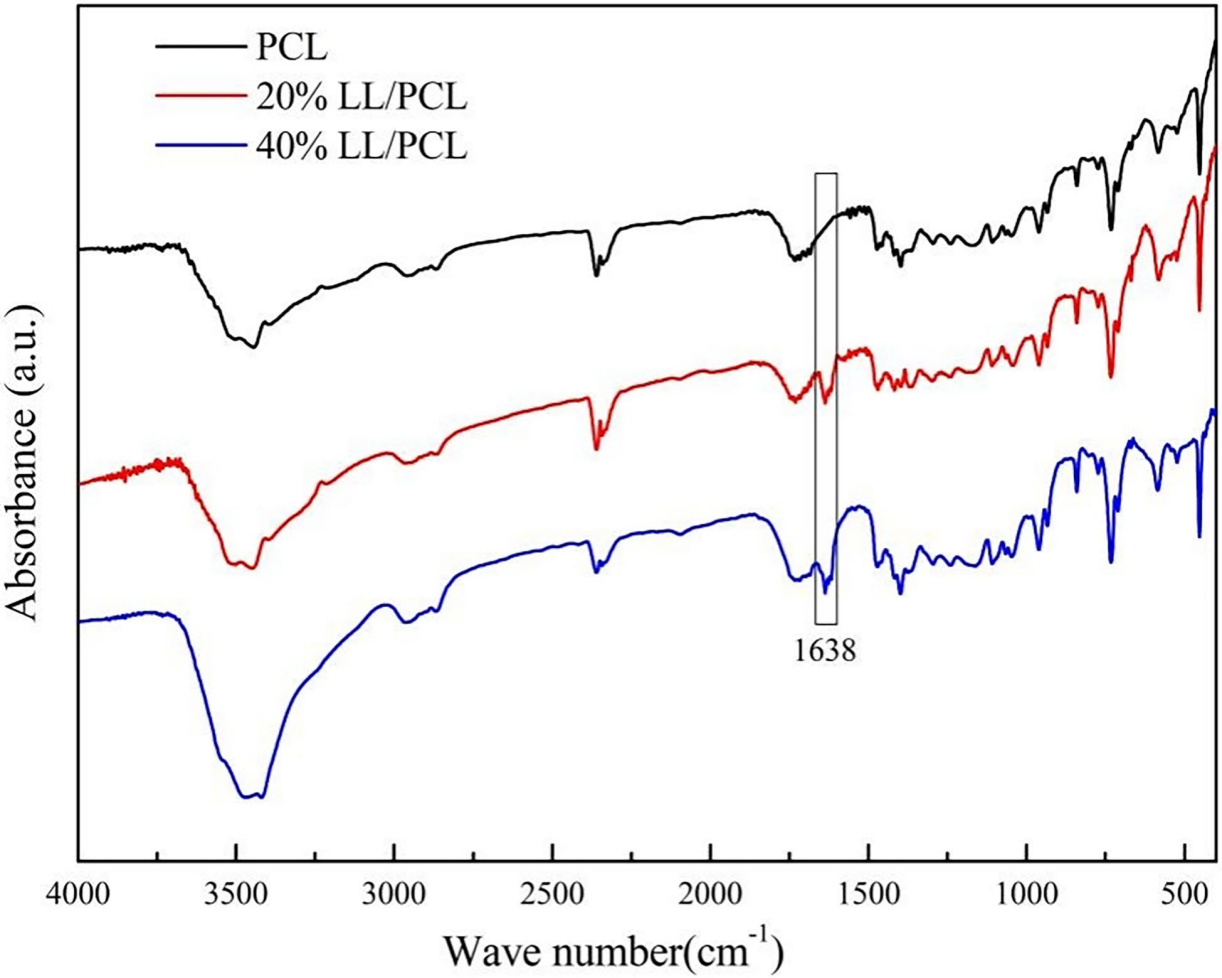
FT-IR patterns of LL/PCL fibrous membranes.

The FT-IR spectra of the LL/PCL fiber membranes are shown in Figure 2. The characteristic absorption peaks of PCL near 1,295 cm^−1^ are stretching vibrations of C▬O and C〓C, the characteristic absorption peak at 1,720 cm^−1^ is the contraction vibration of C〓O in the carbonyl group, and the absorption peaks of the asymmetric and symmetrical telescopic vibrations of CH_2_ are observed at 2,952 and 2,865 cm^−1^, respectively. The fiber membrane has a wide absorption peak in the 3,200–3,600 cm^−1^ region, which is usually attributed to the O▬H stretching vibration. Compared with the uniaxial membrane, the absorption peak of the coaxial spinning film in this region clearly changed with increasing linalool concentration, which may be due to the hydrogen bond between OH and PCL. A new characteristic absorption peak appeared in the spectrum of the coaxial fiber membrane at 1,638 cm^−1^, which represents the stretching vibration of C〓C in linalool, indicating that linalool is present in PCL.

### DSC Analysis

**Figure 3:**
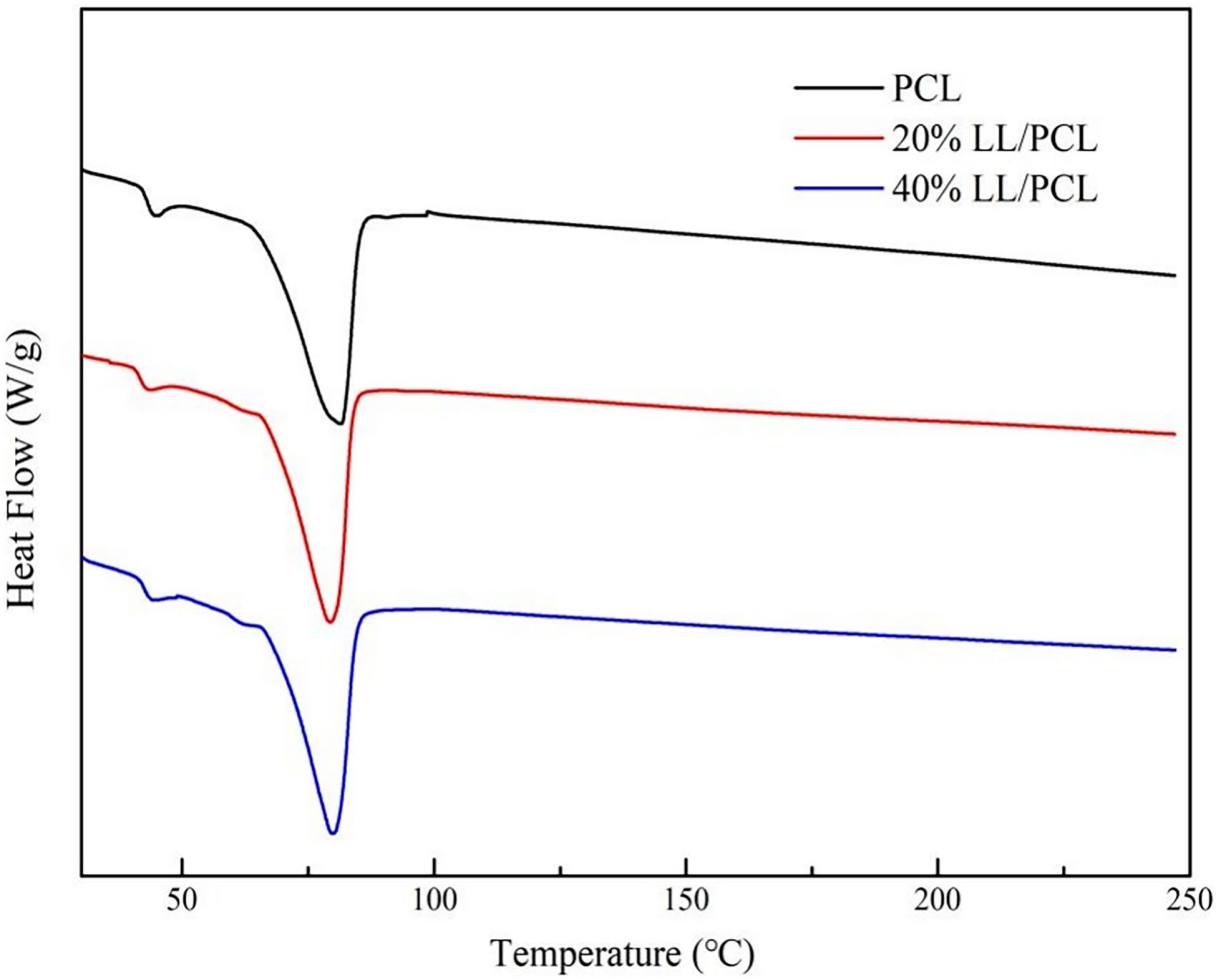
DSC diagrams of LL/PCL fibrous membranes.

The thermal stability of the linalool/polycaprolactone fiber membrane was measured using differential scanning calorimetry. Figure 3 shows two heat absorption peaks in the range of detection for several LL/PCL fiber membranes with different linalool concentrations, where the first peak appearing near 44.37°C may be due to the vaporization of a small amount of *N-N* dimethylformamide solvent present in the fiber membrane. The second peak differed slightly based on the addition of linalool. The heat absorption peak of the PCL fiber membrane appeared at 81.48°C, while that of the coaxial LL/PCL fiber membrane appeared at 79.77°C. This difference may be due to the addition of linalool, which may lead to a slight decrease in the glass transition temperature of the fiber membrane. Generally speaking, there was no obvious difference in the DSC pattern, which means that there is no chemical reaction between the core and wall, and linalool is attached by a simple physical inclusion relationship.

### Thermogravimetry

**Figure 4:**
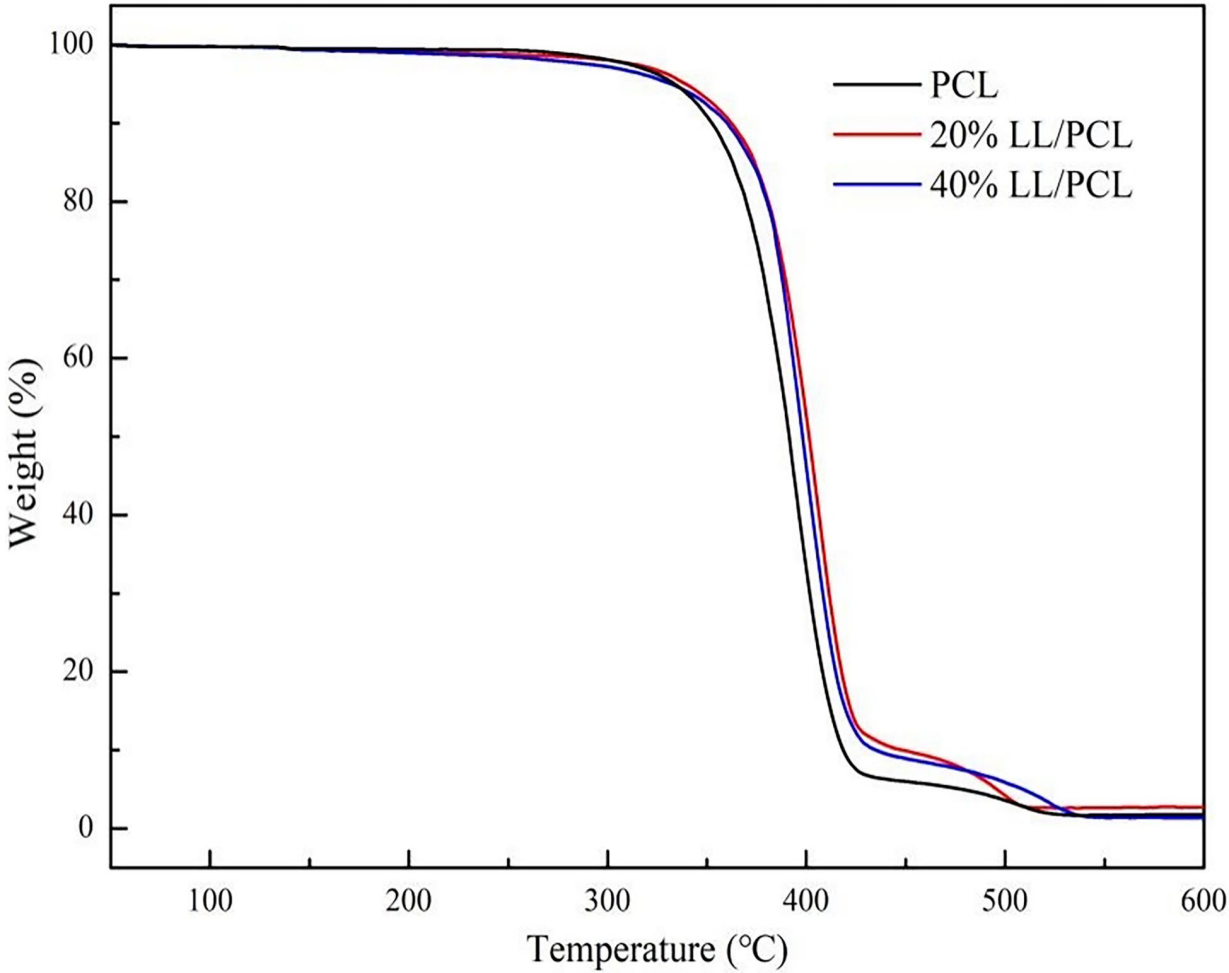
Thermogravimetric curves of LL/PCL fibrous membranes.

Figure 4 shows the thermogravimetric curve of the LL/PCL nanofiber membrane. The thermal degradation profiles of the coaxial and uniaxial electrospun fiber films was similar, with three main stages: the first stage occurs before 320°C, where the thermal weight loss rate of the fiber membrane is approximately 4%. The mass loss in this stage is related to the organic reagents and core materials in the spinning fluid; the PCL-wrapped linalool vaporizes rapidly at this stage due to the external temperature; the second stage is 320°C–430°C, where the thermal weight loss rate of the fiber membrane was above 85%, and the highest thermal weight loss rate of the uniaxial fiber membrane was 89.76%. The mass loss in this stage is mainly caused by the fracture of the ester group (C▬O▬C) on the PCL chain segment. In this stage, the PCL film carbonized and decomposed gradually, and the mass decreased rapidly; the third stage at 430°C–530°C corresponds to complete ashing of the LL/PCL nanofiber membrane. The weight of the fiber membrane after this stage was approximately 2% of the initial mass, and the fiber membrane was completely degraded.

### Analysis of Physical and Chemical Properties of Fiber Membrane

Food packaging materials should have different physical and chemical properties depending on the food type. In the packaging of aquatic products, good hydrophobicity and water barriers have a significant influence on the quality of food. At the same time, packaging materials are also required to have a certain strength to bear the weight and external load of the inner and outer items in the package, and to maintain the performance and integrity of the articles.

**Figure 5:**
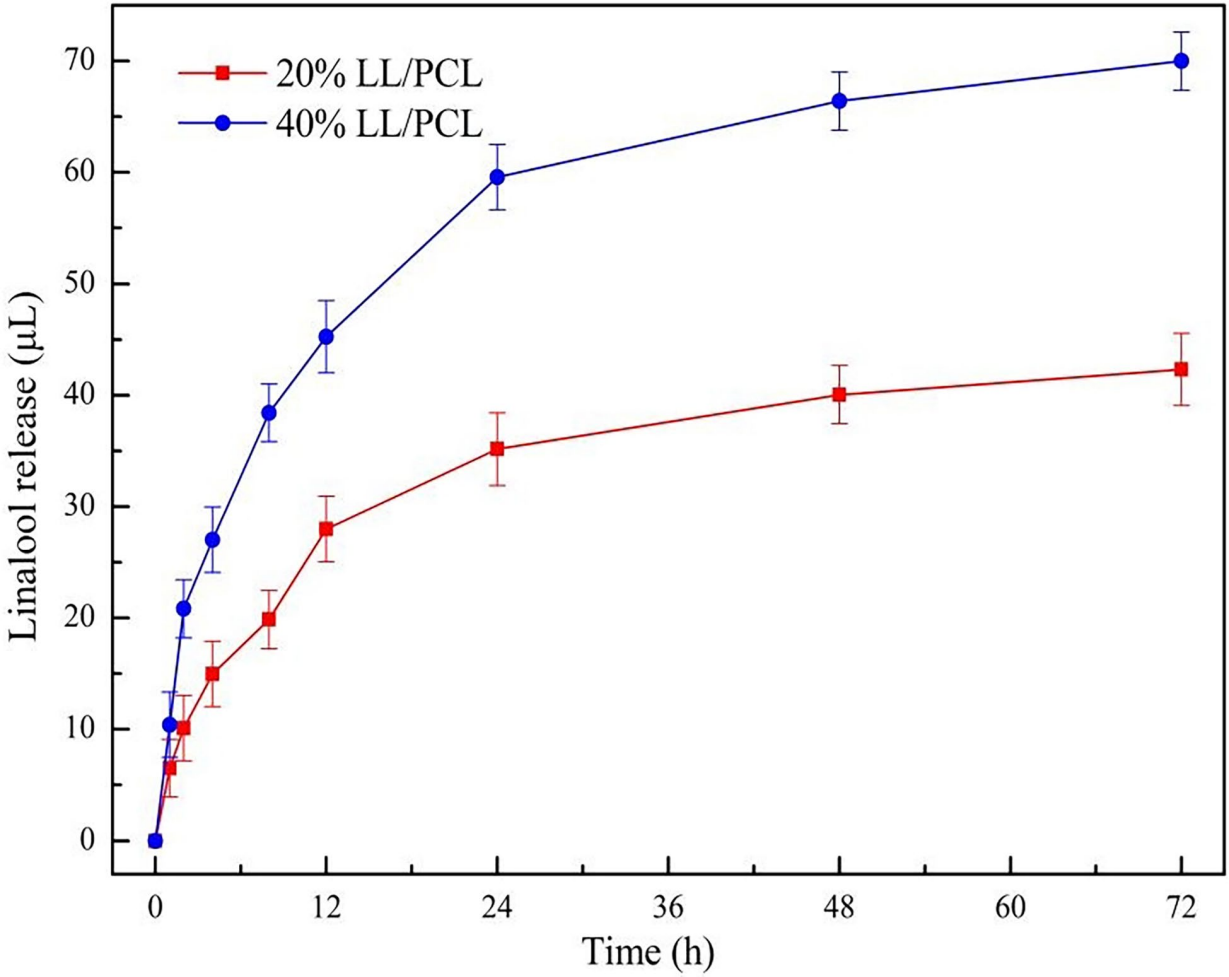
Release curves of LL/PCL fibrous membranes.

The water contact angle of the LL/PCL fiber membranes with different LL concentrations (Table 1) was greater than 90°, demonstrating that the fiber membrane prepared in this experiment is hydrophobic, as clearly illustrated in Figure 5. The water contact angle of the PCL fiber membrane was 113.20 ± 2.52°, the water contact angle of the 20% LL/PCL nanofiber membrane was 127.03 ± 3.67°, and the water contact angle of the 40% LL/PCL nanofiber membrane was 128.37 ± 1.78. The hydrophobicity of the PCL fiber membrane with linalool was enhanced, and the PCL fiber film became more hydrophobic with increasing linalool addition.

**Table 1:** Physicochemical properties of LL/PCL fibrous membranes.

Electrospinning membrane	PCL	20% LL/PCL	40% LL/PCL
WCA (°)	113.20 ± 2.52b	127.03 ± 3.67a	128.37 ± 1.78a
Thickness (mm)	0.052 ± 0.006b	0.063 ± 0.012a	0.065 ± 0.008a
WVT [g/(m^2^·d)]	13.677 ± 0.879c	18.029 ± 1.758b	20.827 ± 1.319a
Ts (MPa)	25.463 ± 0.003a	22.270 ± 1.137b	22.080 ± 0.314b
Eb (%)	129.163 ± 0.283a	113.268 ± 2.456b	110.348 ± 0.841c

The lowercase letters in the table represent significance. *p < 0.05.*

As shown in Table 1, the water vapor transmittance of the PCL film was significantly lower than that of the coaxial LL/PCL fiber membrane due to the addition of linalool. This may be due to the small fiber diameter and low porosity of the single-axis PCL membrane, which leads to a low WVP value. The SEM image in Figure 1 reflects this point well. Kavoosi et al. (2013) also found that the incorporation of high concentrations of thymol in gelatin composite membranes significantly increased the WVP value of the film.

The data in Table 1 show that the tensile strength of the three films exceeded 20 MPa, and the strength of the films is comparable to that of low-density polyethylene (15.2–78.6 MPa). The tensile strength and elongation at break of the PCL films without the core were 25.463 ± 0.003 MPa and 129.163% ± 0.283%, respectively. The results indicate that the diameter of the nanofibers increased, and the porosity increased after PCL loading. Because of the loading of linalool, the close connection between the original PCL molecules was disrupted, and the PCL molecular chain could not be fully stretched. Therefore, the tensile strength of the coaxial fiber membrane was reduced, where the larger the amount of linalool added, the lower the tensile strength of the coaxial fiber membrane.

### Analysis of LL Release From Fiber Membrane

The solubility and compatibility of the drug in the release system are decisive factors for achieving stable drug release. To seal most drugs in polymer fibers and obtain a constant and stable drug release curve, a lipophilic polymer should be chosen for lipophilic drugs, while hydrophilic polymers should be used for hydrophilic drugs. Otherwise, the drug will be located on the surface of the fiber and spread rapidly into the release medium, which will lead to explosive release of the drug, rather than the desired sustained release. Linalool and PCL are hydrophobic substances; therefore, they do not undergo burst release. However, linalool is also released due to the natural degradation of PCL when stored at room temperature for a long time.

Figure 5 shows the release curve for linalool in the LL/PCL fiber membrane. A small burst release was observed in the early stage of sustained release from the fiber membrane, and the release of linalool was significantly increased. This may be because during spinning, the core material is ejected from the wall material, and a small amount of core material is not completely wrapped in the wall; in the early stage, the release of linalool stabilized within 1 days when the flask was violently shaken, and linalool diffused into the reaction system. The release of linalool from the 20% LL/PCL film and 40% LL/PCL film over 3 days was quantified as 42.32 and 69.98 μl, respectively. This is far less than the amount loaded in the LL/PCL fiber film; thus, it is inferred that linalool will not be released completely from the fiber membrane in a short period. Thangaraju et al. (2012) came to similar conclusions in the study of the mechanism of application of curcumin-loaded PLA nanofibers.

### Total Numbers of Colonies

**Figure 6:**
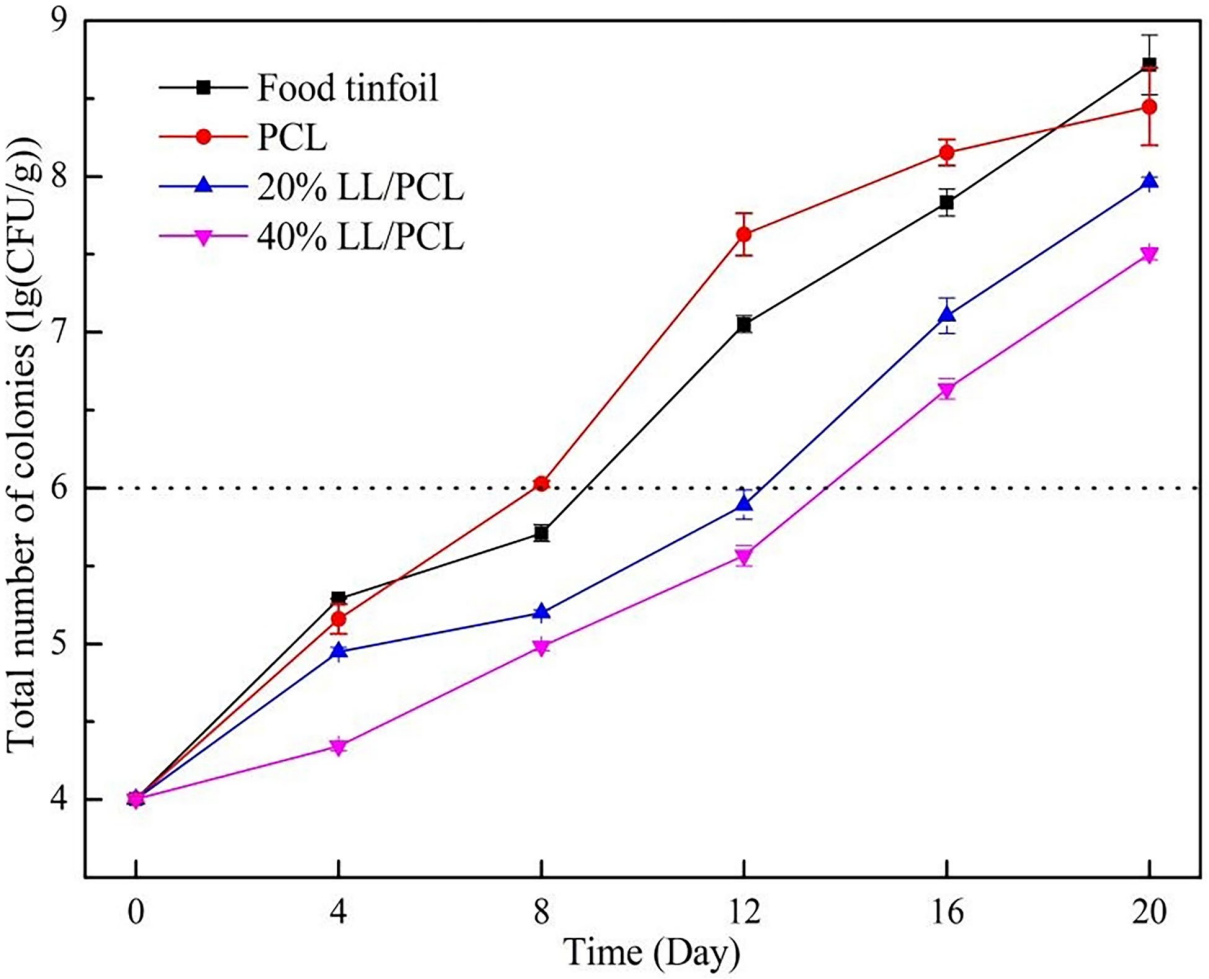
Changes in total microbial count of salmon.

The total number of colonies in salmon slices stored at 4°C is shown in Figure 6. The total number of initial colonies of salmon was approximately 4.004 log CFU/g, where the value mainly depends on the storage status of salmon during transportation (Colin et al., 2019). On the eighth day of storage, the total colonies for the PCL gasket group reached 6.027 lg CFU/g, which is beyond the acceptable range for consumers. The total colonies for the tin paper group was 5.71 lg CFU/g, which is close to the limit. In contrast, the total number of colonies for salmon treated with the 20% LL/PCL fiber gasket and 40% LL/PCL fiber gasket only exceeded the limit on the 16th day, which indicates that the coaxial spinning gasket could extend the shelf-life of salmon, mainly because linalool has a destructive effect on the cell structure of bacteria.

## pH During Storage

**Figure 7:**
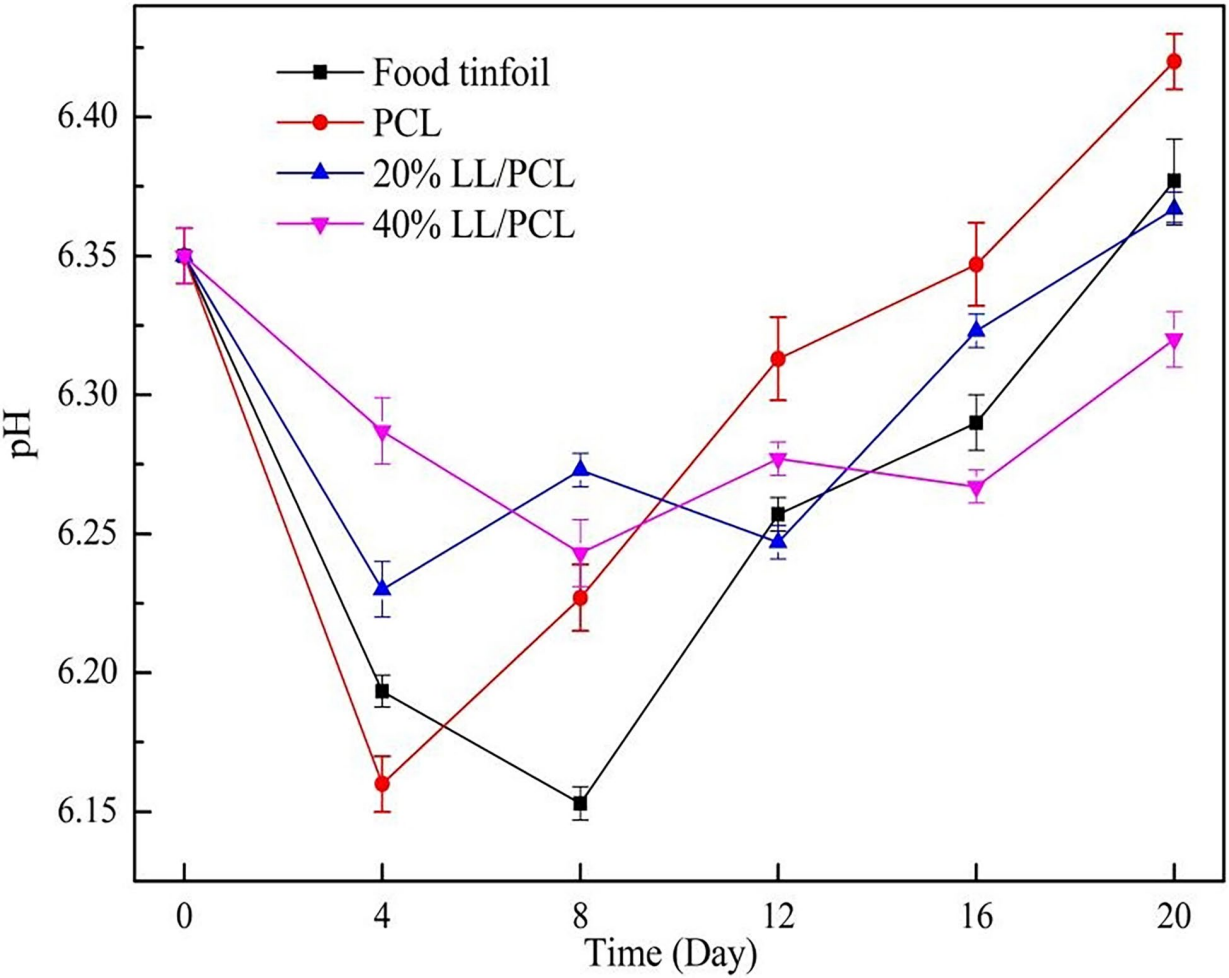
Changes in pH of salmon.

The pH changes of salmon during storage are shown in Figure 7. In the early storage period, because of the role of endogenous enzymes in fish, a large number of acid substances are produced, and the pH of fish meat decreases rapidly, after which the spoilage microorganisms in fish meat increase gradually and are affected by autolyase, resulting in a large number of amine substances, which leads to a gradual increase in the pH (Bhanu et al., 2016). The PCL gasket group reached the lowest pH on the 4th day, and the food tin paper group reached the lowest pH on the eighth day, after which the pH gradually increased to 6.15–6.45. The results show that for the co-axial spinning gasket group, the pH fluctuated over the storage period, and the fluctuation range was smaller than that of the two control groups. This may be because the coaxial spinning gasket can release linalool for a long time, which inhibits the growth of microorganisms and thus delays the increase in amine alkalinity and thus the rise in pH, suggesting that linalool can slow down the spoilage of salmon.

### Total Volatile Basic Nitrogen

**Figure 8:**
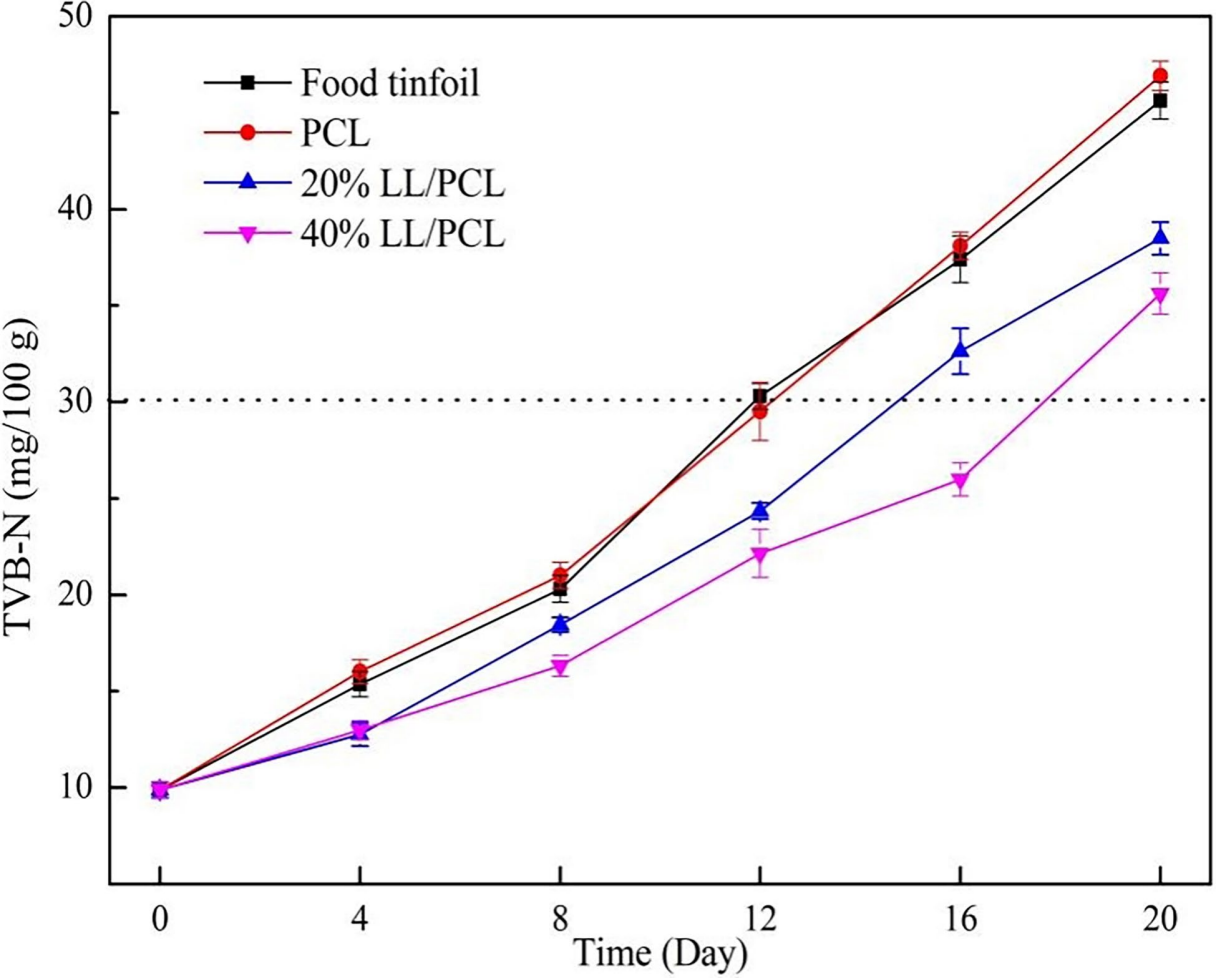
Changes in TVB-N of salmon.

The TVB-N is an important index for assessing the freshness of aquatic products (El et al., 2021). During storage, the protein of aquatic products is decomposed by microorganisms to produce alkaline nitrogen-containing substances, such as ammonia and amine. A TVB-N value exceeding 30 mg/100 g indicates complete spoilage and that the meat cannot be accepted by consumers. As shown in Figure 8, the PCL gasket group and food tin paper group reached this limit on the 12th day of storage, indicating that the fish meat at this time could not be consumed by consumers. The TVB-N value for the 20% LL/PCL coaxial fiber gasket group was 24.347 mg/100 g on the 12th day, which was still in the range of three-stage freshness. The TVB-N value for the 40% LL/PCL coaxial fiber gasket group was 25.987 mg/100 g on the 16th day. This is mainly due to Linalool inhibited the growth of microorganisms, thus reducing the rate of microbial decomposition of salmon, reducing the rate of protein breakdown in the fish, producing less nitrogenous material and ultimately leading to slow decrease in TVB-N content in the 20% LL/PCL and 40% LL/PCL groups.

### Thiobarbituric Acid Reactive Substances

**Figure 9:**
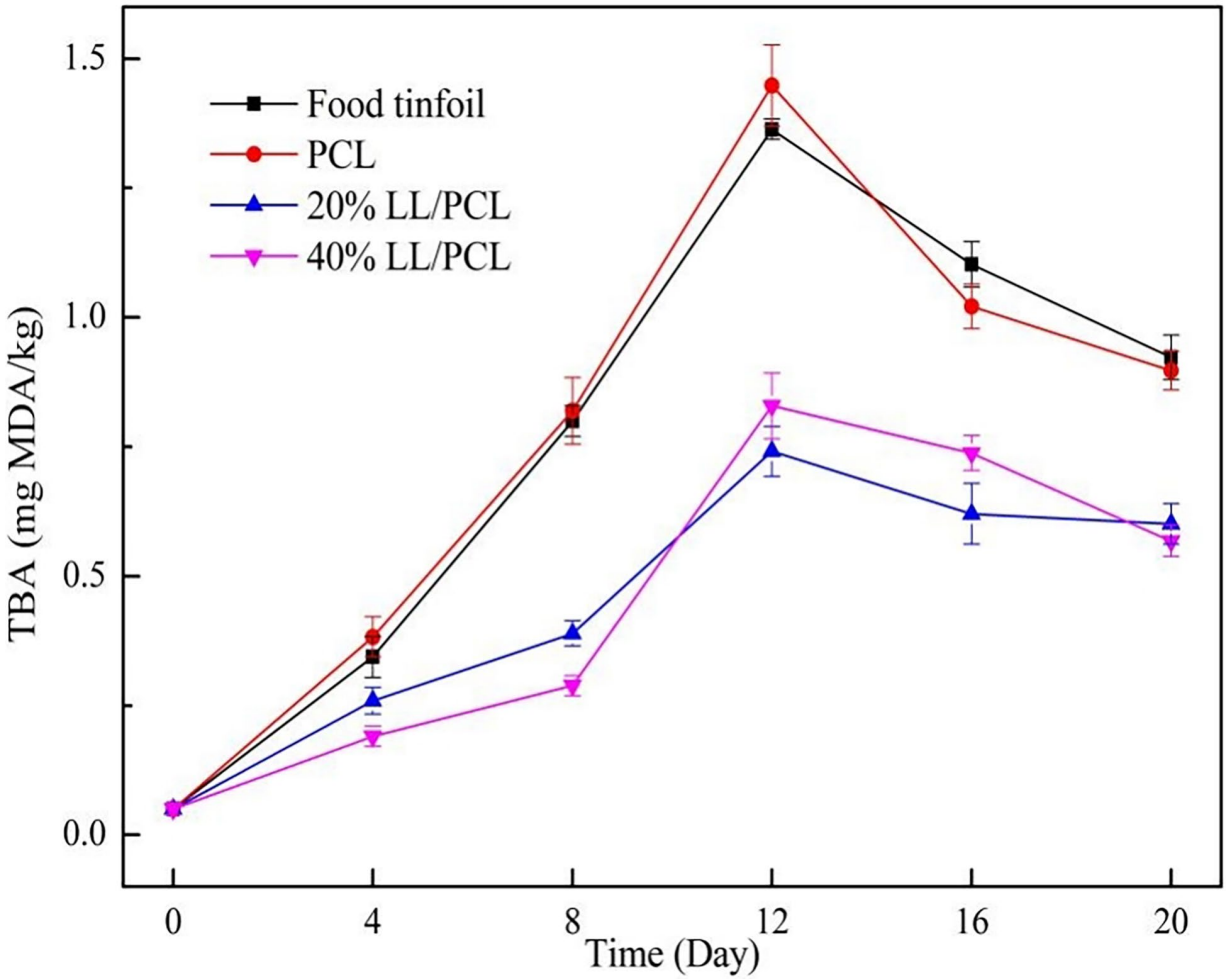
Changes in TBARS value of salmon.

During storage, lipid oxidation in fish is mainly caused by enzyme-induced oxidation and self-oxidation. These two kinds of oxidation can be induced by metal ions, free radicals, fatty oxidase, peroxidase, and microbial enzymes. Lipase and phospholipase are produced by some cold-ophilic bacteria, leading to an increase in free fatty acids and further oxidation (Xu et al., 2020). The TBARS value is an important index for measuring the degree of fat oxidation in aquatic products, as TBARS can react with malondialdehyde (MDA) produced by lipid oxidation. From Figure 9, the initial TBARS value of salmon was 0.05 mg/kg, which increased rapidly in the early storage period. On the 12th day, the TBARS values for the PCL gasket group and food tin paper group were 1.36 and 1.45 mg/kg respectively, while for the group with linalool, the TBARS increased from 0.05 to 0.80 mg/100 g, indicating that the addition of linalool could inhibit the automatic oxidation and hydrolysis of salmon. In the late storage period, the TBARS value decreased due to the substrate fat is oxidized into primary oxidation products, and the primary oxidation products are consumed and the content is reduced, which limits the generation of secondary oxidation products.

### Texture Analysis

**Figure 10:**
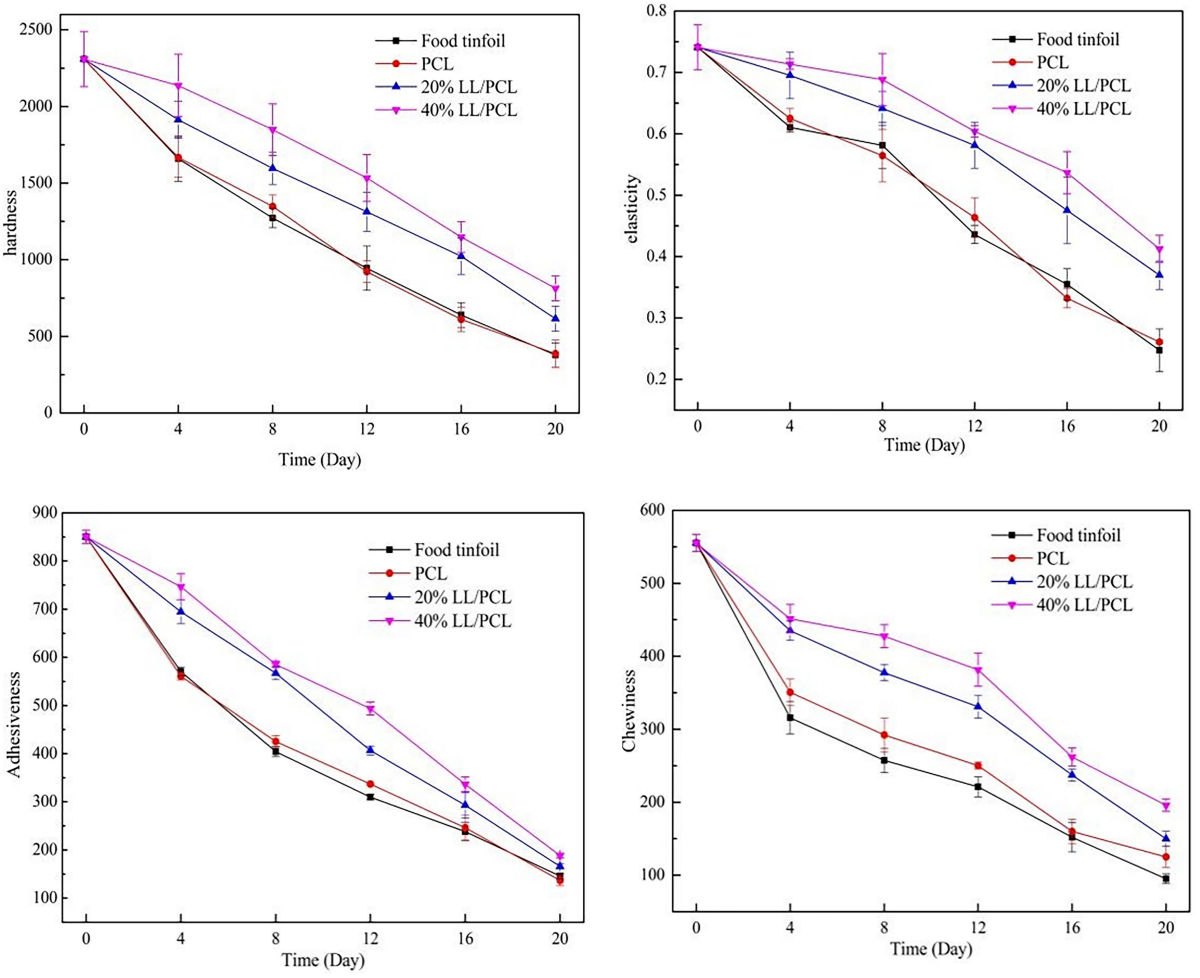
Structural changes of salmon during storage.

During the storage of salmon, due to the growth of spoilage microorganisms and protein denaturation and hydrolysis caused by the autolysis of salmon, the texture index of salmon meat is reduced, and the original textural characteristics are lost (Singh and Benjakul, 2018). Figure 10 shows the textural changes of salmon during low-temperature storage at 4°C, including the chewiness, elasticity, hardness, and adhesiveness. During storage, the hardness of the salmon filets showed a downward trend, which is due to the autolysis of fish after death. The hardness of salmon stored in the two coaxial nanofiber spacer groups containing linalool decreased significantly more slowly than that of the control group, indicating that linalool can maintain the hardness of fish filets. The chewiness, adhesiveness, and elasticity followed a similar trend to that of the hardness. The results showed that linalool was able to inhibit the denaturation and hydrolysis of salmon proteins by inhibiting the growth of microorganisms, thus the rate of decrease of indices such as hardness and elasticity in the 20% LL/PCL and 40% LL/PCL groups was slower, indicating that linalool could maintain a good texture of fish filets and delay the decay of salmon. Cai et al. (2015) also observed that the texture characteristics of turbot, especially the hardness, elasticity, chewiness, and adhesiveness, decreased significantly during cold storage, which is consistent with our results.

### Water Distribution

Low-field nuclear magnetic resonance (LF-NMR) is an effective method for detecting changes in the muscle structure and water mobility in food. It explores the distribution and composition of water in tissues from a microscopic point of view. The relaxation time can reflect the chemical osmotic exchange between bound water and free water (Younas et al., 2020).

Table 2 records the changes in the water distribution of salmon during storage. The combined water distribution and storage time did not have a well-defined relationship, but there was an overall trend for the free water to increase with time, where the difference between each group is small. The proportion of unbound water decreased gradually. The percentage of free water increased significantly, and the difference between the experimental group and the control group was significant (*p* < 0.05). This is mainly due to the effect of endogenous enzymes and spoilage microorganisms in the fish body, which leads to destruction of the protein structure of fish meat, resulting in weaker binding ability of the trapped water in the muscle fiber gap; thus, the proportion of trapped water decreases and the proportion of free water increases. These results indicate that linalool can reduce the content of free water, enhance the binding of water to fish tissue, and reduce the breeding of microorganisms.

**Table 2:** Change in water distribution of salmon during storage.

Storage time (days)	Group	0	4	8	12	16	20
Combined water (%)	Food tinfoil	10.95 ± 1.69^aBC^	13.23 ± 2.29^aAB^	11.02 ± 1.59^bBC^	10.57 ± 1.84^aC^	13.89 ± 2.30^aAB^	14.27 ± 0.49^aA^
	PCL	10.95 ± 1.69^aB^	14.40 ± 2.05^aA^	13.49 ± 1.28^aA^	10.52 ± 0.91^aB^	12.59 ± 2.20^aAB^	14.02 ± 2.77^aA^
	20% LL/PCL	10.95 ± 1.69^aB^	12.36 ± 1.73^bAB^	11.92 ± 2.09^abB^	12.23 ± 1.90^aAB^	13.13 ± 1.34^aAB^	14.20 ± 1.03^aA^
	40% LL/PCL	10.95 ± 1.69^aC^	13.65 ± 1.41^aAB^	10.63 ± 1.19^bC^	12.43 ± 2.54^aBC^	13.16 ± 1.61^aAB^	14.96 ± 1.54^aA^
Immobilized water (%)	Food tinfoil	83.96 ± 1.40^aA^	78.65 ± 1.89^bB^	77.57 ± 0.86^bcB^	72.65 ± 1.54^bC^	69.15 ± 0.88^bD^	65.85 ± 1.72^bE^
	PCL	83.96 ± 1.40^aA^	78.65 ± 1.87^bB^	77.38 ± 1.36^cB^	73.00 ± 1.32^bC^	69.80 ± 1.11^bD^	65.54 ± 2.02^bE^
	20% LL/PCL	83.96 ± 1.40^aA^	81.24 ± 1.89^aB^	79.33 ± 1.86^abB^	75.99 ± 2.52^aC^	71.19 ± 0.99^bD^	67.62 ± 2.62^abE^
	40% LL/PCL	83.96 ± 1.40^aA^	82.19 ± 1.59^aAB^	80.17 ± 1.80^aB^	77.23 ± 1.95^aC^	73.99 ± 1.85^aD^	69.18 ± 2.77^aE^
Free water (%)	Food tinfoil	5.09 ± 2.00^aE^	8.12 ± 2.74^aD^	11.40 ± 2.13^aC^	16.78 ± 1.56^aB^	16.96 ± 1.99^aB^	19.87 ± 1.85^aA^
	PCL	5.09 ± 2.00^aD^	6.95 ± 2.65^abCD^	9.13 ± 2.32^aC^	16.48 ± 1.74^aB^	17.61 ± 2.34^aB^	20.44 ± 2.80^aA^
	20% LL/PCL	5.09 ± 2.00^aD^	6.39 ± 2.33^abCD^	8.75 ± 2.91^aC^	11.78 ± 2.72^bB^	15.68 ± 0.84^aA^	18.19 ± 1.70^abA^
	40% LL/PCL	5.09 ± 2.00^aD^	4.16 ± 0.68^bCD^	9.20 ± 2.20^aC^	10.33 ± 3.63^bBC^	12.85 ± 1.15^bB^	15.86 ± 2.23^bA^

*±Standard deviation; different small letters in the same column showed significant difference between groups (p < 0.05); and different capital letters on the same line indicated significant difference within the group (p < 0.05)*.

### Electronic Nose

An electronic nose is a gas sensor array that simulates the human olfactory system and is able to recognize a sample odor and identify the overall odor composition of the sample by drawing the gas response curve. After the response value is obtained, principal component analysis (PCA) is performed. This analysis method can reduce the dimensions of multidimensional data and extract the core elements of the data.

**Figure 11:**
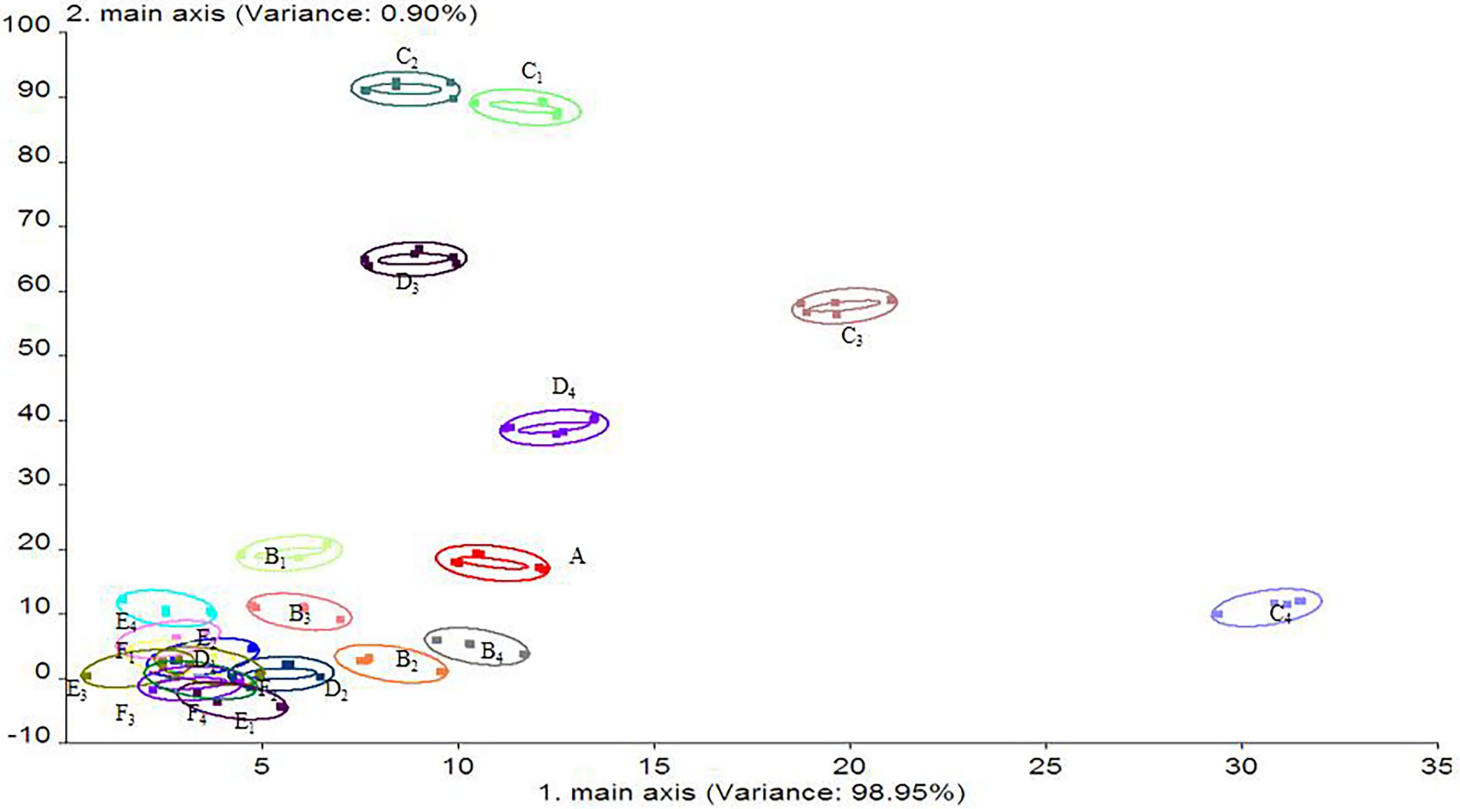
Principal component analysis of salmon flavor. A, B, C, D, E, and F represent 0, 4, 8, 12, 16, and 20 days salmon samples, respectively; subscript 1 represents the food tinfoil group; subscript 2 represents the PCL group; subscript 3 represents 20% LL/PCL group; subscript 4 represents 40% LL/PCL group.

As shown in Figure 11, the contribution rates of the first and second principal components of the stored salmon odor were 98.95% and 0.90%, respectively, and the total contribution rate was 99.85%, indicating that these two principal components can reflect the overall information regarding the volatile odor of salmon during storage (Duan et al., 2021). The results showed that the volatile components of the salmon samples subjected to different treatments were similar, but the odor changed slightly. The 20% LL/PCL group and 40% LL/PCL group showed positive changes along the PC1 axis on the 8th day. These groups differed in that the change along the PC2 axis was less pronounced for the 20% LL/PCL group than for the food tinfoil group and PCL group. On the 12th day of storage, a negative change along the PC1 axis and positive change along the PC2 axis were observed for the 20% LL/PCL group and 40% LL/PCL group, while for the food tin foil group and PCL group, a negative change was observed along the PC2 axis. After 16 days, the volatile components of salmon in the different groups gradually approached the same value and changed little. In summary, it can be seen that the fillets of all groups started to show signs of spoilage at 8 day due to the growth of microorganisms, resulting in a change in the odor of the fillets, but the change in odor was greater in the tin foil and PCL groups than in the 20% LL/PCL and 40% LL/PCL groups, indicating that the 20% LL/PCL and 40% LL/PCL were able to inhibit the growth of microorganisms, thus delaying the spoilage of the salmon fillets. Therefore, the freshness of the 20% LL/PCL and 40% LL/PCL groups could be extended to the 12th day.

## Conclusion

A type of LL/PCL nanofiber membrane was prepared by using the coaxial electrospinning method. The morphology, structure, thermal stability, hydrophobicity, mechanical properties, water vapor permeability, and linalool release properties of the nanofiber membrane were investigated, and the sensory score, total bacterial count, pH, TBARS, TVB-N, water distribution, textural characteristics, and aroma (by analysis of the volatiles using an electronic nose) of salmon slices during storage were detected and analyzed. The results show that the micromorphology of the fiber membrane was good, with a concentrated diameter distribution. Compared with the PCL membrane without the core material, the diameter of the coaxial nanofiber membrane with linalool addition increased, the hydrophobicity and WVP were enhanced, but the tensile strength and elongation at break decreased slightly. There were no significant differences in the thermal stabilities of the three membranes. Linalool was encapsulated in the PCL fiber by a simple physical embedding method and could be released over a long time in a sustained-release manner. The results showed that tin foil and PCL had no significant effect on the freshness of salmon, while the LL/PCL electrospun fiber gasket supplemented with linalool could effectively prolong the shelf-life of salmon and retard the decay of salmon by releasing linalool.

## Data Availability Statement

The raw data supporting the conclusions of this article will be made available by the authors, without undue reservation.

## Author Contributions

All authors listed have made a substantial, direct, and intellectual contribution to the work and approved it for publication.

## Funding

This study was supported by the National Key Research and Development Program of China (nos. 2018YFD0400601 and 2019YFD0901702) and the Opening Foundation of University-Enterprise Alliance of Food Industry in Liaoning Province (2018LNSPLM0104).

## Conflict of Interest

The authors declare that the research was conducted in the absence of any commercial or financial relationships that could be construed as a potential conflict of interest.

## Publisher’s Note

All claims expressed in this article are solely those of the authors and do not necessarily represent those of their affiliated organizations, or those of the publisher, the editors and the reviewers. Any product that may be evaluated in this article, or claim that may be made by its manufacturer, is not guaranteed or endorsed by the publisher.

## Abbreviations

DCM: Dichloromethane
DSC: Differential scanning calorimetry
FT-IR: Infrared spectroscopy
LL: Linalool
LF-NMR: Low-field nuclear magnetic resonance
MDA: Malondialdehyde
DMF: *N,N*-dimethylformamide
PCL: Polycaprolactone
PCA: Principal component analysis
SEM: Scanning electron microscopy
TS: Tensile strength testing
TPA: Texture profiling analysis
TG: Thermogravimetric analysis
TBARS: Thiobarbituric acid reactive substances
TVB-N: Total volatile basic nitrogen
WVP: Water vapor transmission rate
